# The Effects of Mindfulness on Persons with Mild Cognitive Impairment: Protocol for a Mixed-Methods Longitudinal Study

**DOI:** 10.3389/fnagi.2016.00156

**Published:** 2016-06-28

**Authors:** Wee Ping Wong, Craig Hassed, Richard Chambers, Jan Coles

**Affiliations:** ^1^Department of General Practice, School of Primary Health Care, Monash UniversityMelbourne, VIC, Australia; ^2^Mental Health Programs, Campus Community Division, Monash UniversityMelbourne, VIC, Australia

**Keywords:** mindfulness, meditation, mild cognitive impairment, mild neurocognitive disorder, cognitive function, psychological health, functional abilities, activities of daily living

## Abstract

**Introduction:** Mild cognitive impairment (MCI) not only negatively impacts upon a person's life, but it is also seen as an intermediate stage on the progression to Alzheimer's Disease (AD), and therefore warrants early intervention. However, there is currently no effective pharmacological treatment approved for MCI. There is a paucity of evidence that non-pharmacological interventions such as cognitive training could result in improvements in the daily activities functioning of persons with MCI. Growing evidence has shown that mindfulness meditation increases gray matter volume and concentration in brain regions such as the hippocampus and prefrontal cortex, strengthens brain functional connectivity, and enhances psychological well-being which could be beneficial to counteract the memory and cognitive decline of MCI.

**Aims:** We aim to quantitatively investigate whether mindfulness practice can improve the cognitive function, psychological health, mindfulness and functional abilities in activities of daily living of the MCI participants over time; the relationship between the amount of mindfulness practice and degree of improvement in these health outcomes; and the differential effects and interactions of both formal and informal mindfulness practices. We will also qualitatively address the issues about the MCI participants' and familiar support persons' engagement with the program, the nature of group interactions, their program experience, their perceived effects and expectations of mindfulness practice, and the challenges encountered in practicing mindfulness.

**Methods:** Our study is one of the first mixed-methods longitudinal studies with a 1-year follow-up using a pre- and post-intervention design. It involves the MCI participants and their familiar support person in a customized 8-week group-based mindfulness training program. The outcome measures will use the Montreal Cognitive Assessment, Depression Anxiety Stress Scales, Freiburg Mindfulness Inventory and Bayer Activities of Daily Living Scale. The qualitative methods will include participant observation during the program and semi-structured interviews at post-intervention and 1-year follow-up.

**Significance:** This customized MCI group mindfulness training program presents as a promising and feasible non-pharmacological therapeutic intervention option for MCI and a possible preventive strategy for Alzheimer's disease. This study has been registered in the Australian New Zealand Clinical Trials Registry (ANZCTR) (URL: https://www.anzctr.org.au/Trial/Registration/TrialReview.aspx?id=366695) and allocated the ACTRN: ACTRN12614000820606.

## Introduction

According to the World Health Organization, 47.5 million people worldwide have dementia with an annual incidence of 7.7 million new cases every year. This number is projected to increase to 75.6 million people by 2030 and to 135.5 million people by 2050 (WHO, 2016). Notwithstanding the impact of dementia on the families and caregivers of persons with dementia, the economic burden of dementia was estimated to be US$604 billion in 2010, equivalent to 1% of the worldwide gross domestic product (WHO, 2016). Although dementia is not a natural phase of normal aging, the majority of people with dementia are older people (AIHW, 2015). It has been estimated to be the developed world's third largest burden of disease after depression and ischemic heart disease by the year 2030 (Mathers and Loncar, 2006).

Alzheimer's disease (AD) is the most common form of dementia and comprises 60–70% of cases (WHO, 2016). Mild cognitive impairment (MCI) refers to the transitional state of cognitive changes between normal aging and AD (Petersen et al., 2001). The clinical and cognitive criteria for MCI evaluate negative changes in the individual's cognition compared to his/her previous level. These include objective evidence of impairment in one or more cognitive domains (Albert et al., 2011). One of the most commonly implicated cognitive domains is episodic memory–that is, “conscious recall of events and verbal/visual materials, measured by the ability to recall lists of words or recognize faces both immediately and after a delay” (Henry et al., 2011). Preservation of independence in functional abilities and non-diagnosis of dementia are also important for a diagnosis for MCI (Albert et al., 2011).

Each year, approximately 10–15% of MCI patients converted to AD, compared to the 1–2% annual conversion rate of healthy controls (Petersen et al., 1997, 2001). The MCI sub-clinical population is therefore possibly in “a transitional zone between normal cognition function and clinically probable AD” (Winblad et al., 2004), and should be identified and targeted for early therapeutic intervention.

The risk factors for dementia are essentially the same as those for heart disease (Chen et al., 2014). Preventive approaches are important particularly in light of the fact that while several drugs are used for treating AD, there is currently no effective pharmacological treatment approved for MCI. Studies on pharmacological treatments using cholinesterase inhibitors that are approved by the United States Food and Drug Administration (FDA) and the Australian Therapeutic Goods Administration (TGA), such as donepezil (Aricept®), rivastigmine (Exelon®) and galantamine (Razadyne®, Galantyl®, Reminyl®), on MCI patients to provide short-term benefits for mild to moderately severe AD, have produced mixed evidence with inconclusive results on cognitive improvement or progression from MCI to AD (Vega and Newhouse, 2014).

One other factor contributing to the risk of MCI and AD is poor sleep (Tsapanou et al., 2015). Not only has this been associated with a higher risk of AD but also poorer clearance of beta-amyloid from the brain at night (Mander et al., 2015; Sprecher et al., 2015). Mindfulness training has been found to improve sleep in older adults, suggesting another possible link between mindfulness, MCI and AD (Winbush et al., 2007; Black et al., 2015).

Cognitive training is one of the interventions used in the management of MCI (Henry et al., 2011). However, one Cochrane review concluded that due to the inadequate quality of evidence, the limited effects of performance gains derived from cognitive interventions could not be attributed to specific training effects as these improvements did not exceed those in active control conditions (Martin et al., 2011). Another Cochrane review simply found no significant gains from cognitive training (Bahar-Fuchs et al., 2013). More importantly, the few individual studies that found preliminary evidence of potential benefits derived from cognitive training have not established whether the effects were generalized to the daily functioning of persons with MCI. In light of this, there is a need for therapeutic interventions that specifically target risk factors that could be transferrable to activities of daily living (ADL) for persons with MCI.

Mindfulness can be described as a way of training attention and fostering awareness. This can be done through formal attentional training (mindfulness meditation) or informal practice and applications by being more attentive and engaged in daily life. When unmindful, distracted and inattentive, the brain switches into default mode which has been associated with poor mental health (Brewer et al., 2011) as well as higher amyloid-beta deposition (Simic et al., 2014). Mindfulness training has been found to switch off default mode (Brewer et al., 2011).

Studies have also shown a positive association between amount of meditation practice and meta-cognitive awareness (Garland et al., 2009), attentional performance and cognitive flexibility (Moore and Malinowski, 2009). Early intervention with mindfulness training is therefore a feasible and efficacious non-pharmacological therapeutic intervention option that may improve the cognitive function of persons with MCI and prevent progression to AD. Indeed, there is already growing evidence to suggest that meditation may be a potentially useful and affordable treatment approach for enhancing cognition and memory in patients with neurodegenerative diseases (Newberg et al., 2014). The positive effects of meditation on cognition and dementia risk reduction may be achieved holistically through multiple pathways such as producing neuroprotective effects from less stress-induced cortisol secretion with higher brain-derived neurotrophic factor (BDNF) levels; enhancing lipid profiles and reducing oxidative stress, thereby reducing risk for cerebrovascular/age-related neurodegenerative diseases; and reinforcing neuronal circuits and boosting cognitive reserve (Xiong and Doraiswamy, 2009). Moreover, “studies have demonstrated the effects of mindfulness on enlarging gray matter volume (Pagnoni and Cekic, 2007; Luders et al., 2009), increasing gray matter concentration (Hölzel et al., 2008; Vestergaard-Poulsen et al., 2009; Hölzel et al., 2011; Singleton et al., 2014), strengthening brain functional connectivity (Brewer et al., 2011; Jang et al., 2011; Hasenkamp and Barsalou, 2012; Taylor et al., 2013; Wells et al., 2013b), and enhancing psychological well-being (Singleton et al., 2014), thus suggesting the potential benefits of mindfulness on MCI” (Wong et al., 2015). Mindfulness can thus prevent the “tissue volume loss in the hippocampus and posterior cingulate/precuneus (Buckner et al., 2005; Beason-Held, 2011; Fotuhi et al., 2012; Wang et al., 2013), gradual loss of gray matter (Thompson et al., 2003), reduced functional connectivity in the default mode network (Sorg et al., 2007; Gili et al., 2011; Hafkemeijer et al., 2012; Wang et al., 2013), and high chronic psychological distress (Wilson et al., 2007) that are all implicated in the memory and cognitive decline observed in MCI and AD” (Wong et al., 2015).

Two small pilot clinical studies have been conducted on persons with MCI and dementia using the Mindfulness-Based Stress Reduction (MBSR) course. In the first study, a preliminary assessment of the feasibility of eight 2-h weekly MBSR sessions and one mindfulness retreat day that was conducted using a pilot randomized trial with a small sample of 14 MCI adults, found a non-statistically significant trend toward improvement of cognition and well-being (Wells et al., 2013a). The second pilot study involving 12 persons with dementia and 8 carers again found non-statistically significant improvements in mental well-being after attending eight 2.5-h weekly MBSR course but a non-significant decrease in well-being between post-intervention and 3-month follow-up. Even so, qualitative analyses from course observations and interviews showed that some participants with dementia were able to learn mindfulness and experienced subjective improvements in several quality of life dimensions (Leader et al., 2013).

A key methodological limitation in these studies may be that the generic content of the MBSR course was not customized to the specific needs of the participants with cognitive impairment. There is a lack of longer-term monitoring of the longitudinal effects of mindfulness practice on persons with MCI. Another limitation is the failure to achieve statistical significance because of the small sample sizes in these studies. Finally, the main focus of the MBSR course is on formal mindfulness practice (meditation) and may have insufficiently emphasized the informal mindfulness practice in daily living activities, for example (e.g.,) by incorporating informal mindfulness practice into everyday activities and daily experiences. Such informal mindfulness practice can be tied in with existing ADL (e.g., by encouraging people to eat, travel and communicate mindfully) to promote sustained mindfulness outside of meditation sessions. It would also enhance engagement with ADL, significantly increasing cognitive stimulation throughout the day, which could be assumed to be beneficial for persons with MCI. There is preliminary evidence for this, such as Landau et al.'s study which reported increased cognitive activity such as reading, writing and playing games particularly in early- and middle-life, may directly reduce the beta-amyloid before the onset of AD, as shown that older individuals with high cognitive activity had beta-amyloid comparable to the young controls, while those with low cognitive activity had similar beta-amyloid as the AD patients (Landau et al., 2012).

Overall, the above studies suggest that a longitudinal study into a mindfulness training program that incorporates both the formal (mindfulness meditation) and informal (application in daily living activities) practices of mindfulness, and that is customized to the needs, health outcomes and level of understanding of persons with MCI, is warranted. The proposed study will meet this need.

### Study design

This study adopts a non-randomized pre-/post-intervention design. Persons with a clinical diagnosis of MCI from treating health professionals will receive an 8-week group-based mindfulness training program which is customized to their needs, health outcomes and level of understanding. A follow-up assessment will be conducted one year after the program completion to measure the longitudinal effects of mindfulness practice on persons with MCI over time.

### Research questions

Our hypotheses are:

- That mindfulness practice will improve the cognitive function, psychological health, mindfulness and ADL functioning of persons with MCI.
- The degree of these improvements in cognitive function, psychological health, mindfulness and ADL functioning will correlate positively with the amount of mindfulness practice.

To test these hypotheses, the following quantitative research questions were formulated:

- Does mindfulness practice improve the cognitive function, psychological health, mindfulness and ADL functioning of persons with MCI?
- Does the degree of improvement in cognitive function, psychological health, mindfulness and ADL functioning vary with the amount of mindfulness practice?
- How much of any observed improvement is due to formal mindfulness practice and how much is due to informal applications of mindfulness to ADL?

Further, this study aims to address the following qualitative research questions:

- How do the MCI participants and familiar support persons (FSPs) engage with the mindfulness training program?
- What is the nature of group interactions between the Mindfulness Trainer and the MCI participants/FSPs and amongst the MCI participants/FSPs during the program?
- What is the mindfulness training program experience for the MCI participants and FSPs (e.g. was it enjoyable, satisfactory, stimulating and useful)?
- What perceived effects do the mindfulness training program and mindfulness practice generally have on the health and life of MCI participants and FSPs?
- What are the challenges faced by MCI participants in implementing and maintaining mindfulness practice?
- Do the expectations of the MCI participant and his/her FSPs about mindfulness practice match with each other?

### Study eligibility criteria

To participate in this study, the MCI participants must fulfil the following inclusion criteria:

- Clinical diagnosis of MCI;
- Must be able to give informed consent to participate by signing the Consent Form; and
- At least 60 years old.

The clinical diagnosis and minimum age requirement fit the target sample of participants that this study is investigating on the premise that they must possess the capacity to receive information about their involvement in this study, to consent to the research and to participate in it.

Participants will be excluded based on the following criteria:

- Current active or past significant experience with meditation or yoga;
- Current use of prescribed cognitive intervention or electromagnetic stimulation;
- Acquired/Traumatic brain injury;
- Started new neurological/psychoactive medication within 3 months prior to the first data collection session;
- Current intake of drugs that significantly alter cognition;
- Illicit drug or alcohol abuse or dependence (more than 2 standard units of alcohol at a time and/or more than 7 standard units of alcohol in 1 week) within the previous 5 years;
- Current intake of cholinesterase inhibitors;
- Current severe psychiatric condition *(e.g., bipolar disorder or clinical depression)*; or neurological/cerebrovascular condition; or chronic medical condition *(e.g., advanced stage of cancer, chemotherapy)* that requires intensive medical treatment and monitoring; or advanced cognitive decline;
- Major impairments in eyesight, hearing or upper limb motor movements; or
- English language difficulties.

Exclusion criterion (a) of not having current active or past significant meditation or yoga experience is relevant so as to properly measure the effects of mindfulness on the outcome measures. Exclusion criteria (b) to (f) are necessary to exclude MCI participants currently using prescribed interventions; recently started taking new neurological/psychoactive medication or cognition-altering drugs; or who had suffered brain injury and drug or alcohol abuse due to their possible effects on cognition and behavior. Exclusion criterion (g) of current intake of cholinesterase inhibitors is relevant to screen out persons with severe cognitive impairment. Exclusion criterion (h) is important as this study is investigating the effects of mindfulness on persons with MCI, thus it should not be confounded by other current severe chronic conditions. Exclusion criteria (i) and (j) are relevant to ensure that the MCI participants are able to participate in this study as they will be asked to read, write, listen and speak in English during the program and data collection sessions that include completing cognitive assessments and questionnaires/scales, and communicating in interviews. Information about the potential MCI participants (contact details and relevant personal and health information) and their FSP (name and contact details) may be obtained from the MCI participants' health records for the purpose of this research.

There are no lifestyle restrictions such as physical restrictions, sport participation or dietary restrictions imposed on the MCI participants who may continue to take their regular prescribed medications. However, if their medical condition, treatment or prescribed medications change during the entire duration of study participation, they or their FSP should inform researcher Wee Ping Wong (WW) as soon as possible. In addition, if the MCI participant or his/her FSP takes part in other research studies prior to involvement in this study or intends to participate in other research studies at any subsequent stages during this study duration, they are requested to inform the research team at the earliest opportunity.

### Recruitment

Participation in this study is voluntary with no limitations or conditions imposed and the MCI participants and FSPs are free to withdraw consent from this study at any stage by informing the research team. Their decision whether to take part or not, or to take part and then withdraw, will not affect the MCI participants' routine care, services, medication regimen or relationship with their treating health professionals.

To ensure representativeness of the target population, eligible MCI participants will be recruited within Victoria, Australia through hospitals (including Cognitive, Dementia and Memory Service (CDAMS), specialist clinics, General Practice clinics, Alzheimer's Australia and the general community in accordance with the study eligibility criteria. Staff from the participating CDAMS sites will brief potential MCI participants about this study; inform them that their participation or not in the project will not in any way affect their routine care, services, medication regimen or relationship with treating health professionals. If the staff obtain verbal consent of interested persons with MCI to provide their contact details and relevant personal and health information, and their FSP's contact details to the research team, a referral form will be sent to researcher (WW). Interested persons with MCI who respond to the study advertisement will directly contact researcher (WW) who will brief them about this study over the phone. In both situations, the researcher will liaise with both the person with MCI and his/her FSP to explain the nature of the study participation, confirm the potential MCI participants' study eligibility, send them the Participant Information Sheets and Consent Forms, and arrange the first data collection session. This provides time for them to discuss their study participation and other parties such as General Practitioner (GP) or treating health professional. For interested persons with MCI who are not referred from the participating CDAMS sites to this study, their verbal consent will be obtained for the research team to write to their GP or treating health professional who needs to provide their relevant personal and health information (including their MCI diagnosis details) so that their study eligibility could be established.

At the first data collection session, another opportunity is open for the potential MCI participant and FSP to ask further questions, and seek any clarification about this study and the nature of their involvement. Researcher (WW) will review and discuss this study and the Participant Information Sheets and Consent Forms with the potential MCI participant in the presence of his/her FSP and decide whether he/she is able to:

- Understand the nature of the research and of his/her participation;
- Appreciate the consequences of the participation, including personal consequences;
- Show the ability to consider alternatives, including the option not to participate; and
- show the ability to make a reasoned choice (Lyketsos et al., 2004).

If the potential MCI participant is able to paraphrase the information that has been provided and is aware of his/her rights to refuse or discontinue participation, this will be seen as indicating capacity to consent. Prior to study participation, the potential MCI participant must be able to give his/her free voluntary written informed consent to participate by signing the Consent Form in the presence of his/her FSP who will also confirm his/her capacity to decide study participation and support his/her participation. The FSP will also be asked to provide his/her written informed consent by signing the Consent Form. The MCI participant is recommended to advise his/her GP of his/her decision to participate in this study.

The recruitment of the MCI participant with his/her FSP is preferred but not mandatory. The FSP can be a family member, guardian or person authorized by law, and must be at least 18 years old with no English language difficulties. He/she can confirm the MCI participant's capacity for informed consent, and can provide information about the MCI participant's functional abilities, and demographics, health and lifestyle details. The knowledge of the FSP about the MCI participant is crucial to corroborate the MCI participant's capacity for informed consent and then provide information that could add value to the data. The FSP is required to be present with the MCI participant during the first data collection session to witness the consent process, and is also encouraged to attend the second and third data collection sessions, and the 8-week group mindfulness training program together with the MCI participant.

All MCI participants will be provided with the mindfulness training program manual and a mindfulness practice disc containing guided audio recordings to guide and support them with their home mindfulness practice, and a gift card valued at $50 to compensate them for travel costs. The FSP is encouraged to use these resources and practice mindfulness with the MCI participant as a show of support.

## Materials and methods

This study was approved by the Melbourne Health Human Research Ethics Committee (Project number: HREC/14/MH/324) and will be carried out in accordance with the National Statement on Ethical Conduct in Human Research (2007) to protect the interests of the participants from whom written informed consent will be obtained. This study has been registered in the Australian New Zealand Clinical Trials Registry (ANZCTR) and allocated the ACTRN: ACTRN12614000820606. All the data and information collected for, used in, or generated by this study will be securely stored for 5 years from the date of publication to allow reference and research discussion after which time it will be securely disposed of in accordance with the guidelines required by the Melbourne Health Human Research Ethics Committee.

### Intervention

In addition to the standard care that the MCI participants obtain from their treating health professionals, they will receive the mindfulness training intervention. The intervention will be customized to the needs, health outcomes and level of understanding of MCI participants with a 1-year follow-up period. Participation in the 8-week group mindfulness training program will involve the MCI participants (preferably accompanied by their FSP) attending a 1.5-h group session, once weekly for a total period of 8 weeks. The MCI participants' attendance will be recorded and they are required to attend at least six of the eight sessions to have satisfactorily completed the program.

The program intervention will be designed, customized and facilitated either by a GP Craig Hassed (CH) or a Clinical Psychologist Richard Chambers (RC) who are experienced mindfulness experts and Monash University Mindfulness Consultants. They will be blinded to the identifiable data. The program includes both formal and informal mindfulness practices. Participants will be encouraged to practice formal mindfulness meditation techniques starting with 10 min twice a day and increasing this time up to 20 min twice a day as the program progresses. The formal mindfulness practice involves using techniques such as “body scan” and “breath” meditations to observe and maintain attention on an object of meditation without judgment and reactivity even when one becomes distracted by drifting thoughts or uncomfortable emotions. Informal mindfulness practice involves extending such attentiveness and awareness to engaging daily experiences such as mindful walking and eating. The informal practice will also include mental exercises such as reading, puzzles and language as well as being mindful of daily interactions and other ADL. Participants will also explore how mindfulness can help with attention and memory, ADL functioning, stress, anxiety and depression, emotion management, learning, mental flexibility and problem solving, and sleep with in-class and home practices (see Table 1 for a more detailed program outline).

**Table 1:** **Customized 8-week group-based MCI mindfulness training program outline**.

**Week 1**
• Aims of the program
• Outline the principles and structure of the program, resources and role of Familiar Support Person (FSP)
• Introduction
What mindfulness is and why it can be helpful for MCI
• Importance of mindfulness practice (Formal and Informal approaches)
• In-class mindfulness meditation with guided instructions–e.g., “Body scan” meditation (10 min) and Debrief
• In-class informal mindfulness practice with guided instructions–e.g., mindful eating
• “Questions and Answers” session
• Home practice
Formal mindfulness practice (2 × 10-min “Body scan” meditation daily)
Informal practice in daily life–e.g., mindful eating
Inquiry: Noticing mindfulness vs. default mode and Effects of each
**Week 2**
• In-class “Breath” meditation (I) with guided instructions emphasizing curiosity (5 min)
• Debrief home practice from the last week
• Discussion about Mindfulness, Attention and Memory (Curiosity and Focus)
• In-class “Breath” meditation (II) with guided instructions emphasizing gentleness (10 min)
• Importance of mental exercises
• In-class mental exercises with series of choices–e.g., crossword puzzles, Sudoku, spot the differences etc. and Debrief
• “Comma”
To illustrate that formal practice can be done anytime and anywhere quickly (does not require 10 min)
• Home practice
Formal mindfulness practice (2 × 10-min “Body scan”/”Breath” meditations daily)
Informal practice in daily life–e.g., mindful eating
Mental exercises–e.g., reading, puzzles, language (at least 15 min daily)
**Week 3**
• In-class Mindful listening meditation with guided instructions (5 min)
• Debrief home practice from the last week
• Mindfulness and Activities of Daily Living (ADL) functioning (Part I)–e.g., communications, medication management, use of technology and domestic appliances, cooking, shopping
• In-class mindfulness meditation with guided instructions–“Full stop” (15 min)
• In-class mindfulness exercises with guided instructions–e.g., “Music” and “Communicating with awareness” and Debrief
• Home practice
Formal mindfulness practice (2 × 15-min “Body scan”/”Breath” meditations daily)
Informal practice in daily life–e.g., mindful eating/communications, physical exercises
Mental exercises–e.g., reading, puzzles, language (at least 15 min daily)
**Week 4**
• In-class mindfulness meditation (I) with guided instructions (10 min)
• Debrief home practice from the last week
• Mindfulness and Stress, Anxiety, and Depression
The role of default mental activity in mental health problems
The importance of being in the present moment
• In-class mindfulness meditation (II) with guided instructions (15 min)
• Home practice
Formal mindfulness practice (2 × 15-min “Body scan”/“Breath”/Mindful listening meditations daily)
Noticing stress response, default mode and returning attention to present
Informal practice in daily life–e.g., mindful eating/communications, physical exercises
Mental exercises–e.g., reading, puzzles, language (at least 15 min daily)
**Week 5**
• In-class mindfulness meditation with guided instructions (20 min)
• Debrief home practice from the last week
• Mindfulness and Emotion management
• In-class “Working with distractions” experiment with guided instructions and Debrief
• In-class mindfulness exercises with guided instructions–e.g., “Working mindfully with emotions” and “Compassion/Loving-kindness”
• Home practice
Formal mindfulness practice (2 × 20-min “Body scan”/“Breath”/“Loving-kindness” meditations daily)
Informal practice in daily life–e.g., mindful eating/communications, physical exercises
Mental exercises–e.g., reading, puzzles, language (at least 15 min daily)
**Week 6**
• In-class mindfulness meditation with guided instructions (20 min)
• Debrief home practice from the last week
• Mindfulness and Learning, Mental flexibility, and Problem solving
• In-class mindfulness exercises with guided instructions–e.g., “Multi-tasking–Communicating and Problem-solving”
• Written exercises–To acknowledge distractions/note actual thoughts on the second column/writing pad
• Home practice
Formal mindfulness practice (2 × 20-min “Body scan”/“Breath”/“Loving-kindness” meditations daily)
Informal practice in daily life–e.g., mindful eating/communications, physical exercises
Mental exercises–e.g., reading, puzzles, language (at least 15 min daily)
**Week 7**
• In-class mindfulness meditation with guided instructions (20 min)
• Debrief home practice from the last week
• Mindfulness and ADL functioning (Part II)–e.g., financial management, transportation, driving, use of public transport, walking
• In-class mindfulness exercises with guided instructions–e.g., 15 min of “Mindful walking”
• Home practice
Formal mindfulness practice (2 × 20-min “Body scan”/“Breath”/“Loving-kindness” meditations daily)
Informal practice in daily life–e.g., mindful walking (both as exercise and incidental when traveling)/eating/communications, physical exercises
Mental exercises–e.g., reading, puzzles, language (at least 15 min daily)
**Week 8**
• In-class mindfulness meditation with guided instructions (20 min)
• Debrief home practice from the last week
• Debrief course
Importance of formal mindfulness practice
Importance of incorporating informal mindfulness into day-to-day activities
Mindfulness as a way of life
Mindfulness and MCI/Alzheimer's disease
• Ongoing home practice
Formal mindfulness practice (2 × 20-min “Body scan”/“Breath”/“Loving-kindness” meditations daily)
Informal practice in daily life–e.g., mindful eating/communications/walking, physical exercises
Mental exercises–e.g., reading, puzzles, language (at least 15 min daily)

The MCI participants who could be assisted by their FSP, will be asked to record on their mindfulness practice record forms in the program manual, daily entries of their formal and informal mindfulness practices during the 8-week group mindfulness training program, and weekly entries during the 1-year follow-up. The MCI participants are requested to submit their daily practice record forms weekly during the program and the weekly post-program practice record forms to researcher (WW) on a monthly basis for effective monitoring of their mindfulness practice progress over the follow-up period. Weekly reminders to the MCI participants and/or their FSP will be done by email or phone during the program duration. After the program completion, they will receive monthly reminders by email or phone to follow up with their mindfulness practice progress. They could also contact the researchers should they have questions or concerns at any stage.

It is possible that the mindfulness training program and/or data collection sessions may arouse concerns or anxieties among the MCI participants or FSPs. As the program is customized and facilitated by experienced mindfulness experts, such discomfort is anticipated to be minimal. Measures have been taken to ensure their well-being throughout their study participation/involvement. However, if they become upset or distressed as a result of their participation in the research, they can approach the researchers or their GP. They will also be provided with contact details of telephone support services.

If the MCI participant decides to withdraw, his/her GP or treating health professional will be updated. However, if the FSP decides to withdraw and the MCI participant chooses to remain in this study, discussion will be undertaken with both of them either to find another suitable FSP to continue taking part for the purpose of the continuity of the data collection and analysis, or the MCI participant may continue without the FSP if he/she so wishes. The research team will not collect additional data and information from withdrawn MCI participants and FSPs, although with approval, previous data and information already collected, will be retained to form part of the study results to ensure proper measurement.

### Outcome measures

This study undertakes a mixed-methods approach involving the collection and analysis of quantitative data using cognitive assessments and questionnaires, and of qualitative data collected through participant observation and semi-structured interviews.

#### Quantitative data

The primary outcome is the change in cognitive function assessed by the Montreal Cognitive Assessment (MoCA) that measures the MCI participants' eight cognitive domains of visuospatial/executive, naming, memory, attention, language, abstraction, delayed recall, and orientation over time. The MoCA has excellent sensitivity of 90%, high test-retest reliability, predictive values, content validity and specificity, and good internal consistency (Cronbach's alpha of 0.83; Nasreddine et al., 2005). The MoCA will be administered by researcher (WW) during all three data collection sessions.

The secondary outcomes measured will be changes in psychological health, mindfulness and ADL functioning. The MCI participants will complete the following self-rated questionnaires during all three data collection sessions:

- 21-item Depression Anxiety Stress Scales (DASS-21) that measures psychological health on the axes of depression, anxiety and stress on a continuum of severity over the past week (high internal consistency with respective Cronbach's alphas of 0.94, 0.87, and 0.91; Antony et al., 1998); and
- 14-item Freiburg Mindfulness Inventory (FMI) that measures the experience of mindfulness within the past 7 days (high internal consistency with Cronbach's alpha of 0.86, construct validity and sensitivity to change; Walach et al., 2006).

In addition, another secondary outcome measure of ADL functioning will be assessed using the 25-item Bayer Activities of Daily Living (B-ADL) Scale (Cronbach's alpha above 0.98) which will be completed by the FSP who will rate the MCI participant's functional deficits in the performance of everyday activities (Hindmarch et al., 1998; Erzigkeit et al., 2001).

The MCI participants' adherence of formal and informal mindfulness practices will be measured using the 12-item Mindfulness Adherence Questionnaire (MAQ) which was developed specifically for this study (see Supplementary Material). This questionnaire that will be administered during all three data collection sessions, prompts MCI participants to rate their mindfulness practices over the past week. Further, with general health and lifestyle factors being potential confounding factors, information about the MCI participant's health, physical and social activity, lifestyle activities, dietary intake, alcohol consumption, smoking and demographic details will be collected from the FSP during all three data collection sessions, using a Demographic, Health and Lifestyle (DHL) questionnaire that was adapted from the Australian Longitudinal Study on Women's Health (The Active Australia Survey, 2003; Brown et al., 2008; Seventh Survey for the Women of the 1946–51 Cohort, 2013), the Diet Habits Questionnaire (McKellar et al., 2008) and the Lifestyle Activity Questionnaire (Carlson et al., 2012) for this study (see Supplementary Material).

In the event of the FSP's unavailability during the second and/or third data collection sessions, he/she can return the completed B-ADL Scales and DHL questionnaires using reply paid return envelopes provided.

#### Qualitative data

Qualitative data collection will involve participant observation of the 8-week group mindfulness training program and individual semi-structured interviews conducted separately with the MCI participant and his/her FSP. The recruitment target of which qualitative data saturation with no new discoveries is anticipated to be about 20 MCI participants.

Researcher (WW) will attend all the eight sessions of the group mindfulness training program to conduct participant observation in order to evaluate how the MCI participants and FSPs engage with the program, particularly with the core modules of “attention and memory,” “ADL functioning,” “stress, anxiety and depression,” “emotion management,” “learning, mental flexibility and problem solving,” and to assess the nature of group interactions between the Mindfulness Trainer and the MCI participants/FSPs, and amongst the MCI participants/FSPs during the program. Notes will be recorded during and following the observation for subsequent qualitative data analysis.

Semi-structured interviews will be conducted by researcher (WW) during the data collection sessions at post-intervention (T2) and 1-year follow-up (T3). During each of these two data collection sessions, separate interviews with the MCI participant and his/her FSP will be done after the MCI participant has completed the cognitive assessment and questionnaires/scales. These interviews aim to elicit responses from individual perspectives of the MCI participant and his/her FSP in relation to their own mindfulness training program experience; the perceived effects of the program and mindfulness practice have on the health and life of MCI participants and FSPs; the challenges faced by the MCI participants in implementing and maintaining mindfulness practice; and the expectations of the MCI participants and FSPs about mindfulness practice. The interview questions for the MCI participant at T2 and T3 are appended in Table 2; and the separate set of interview questions for the FSP at T2 and T3 is appended in Table 3. In the event of the FSP's unavailability during T2 and/or T3 data collection sessions, arrangements can be made for the interviews to be conducted over the phone. All the interviews will be recorded on a digital audio recorder to ensure accurate representation of responses, and transcribed verbatim by researcher (WW).

**Table 2:** **The interview questions for the MCI participants at post-intervention (T2) and 1-year follow-up (T3)**.

**Qn**	**Post-intervention (T2)–30 min**	**1-year follow-up (T3)–30 min**
1	Can you please describe your general health for me?	
	I really appreciate you sharing your views.	
2	Can you tell me as much as possible how has the mindfulness training program experience been for you?	
	*[Interviewer's prompts: satisfactory? stimulating? useful?]*	
3	What did you like or enjoy about the program?	
	What did you not like about the program?	
	From your perspective, how can we improve the mindfulness training program experience for future participants?	
4	Can you please tell me how you practice mindfulness?	
	How do you use mindfulness in your day-to-day life?	
5	Can you please tell me what has helped or hindered your mindfulness practice?	
6	Can you please tell me in as much detail as possible how you have been since practicing mindfulness?	
	*[Interviewer's prompts: cognitive function, psychological health, functional abilities and quality of life]*	
7	From your perspective, how did your mindfulness practice influence your health and life?	
	*[Interviewer's prompts: cognitive function, psychological health, functional abilities and quality of life]*	
8	Do you think you would continue to practice mindfulness?	
	How long would you continue?	
9	Do you need any ongoing support and reminders to maintain regular mindfulness practices after the program?	Do you need any ongoing support and reminders to continue regular mindfulness practices from now on?
	If yes, what kind of ongoing support and reminders?	If yes, what kind of ongoing support and reminders?
10	What advice would you give to other people like yourself about undertaking mindfulness training?	What advice would you give to other people like yourself about practicing mindfulness?
11	Can you please describe the role of your familiar support person in the mindfulness training program and outside the program?	Can you please describe the role of your familiar support person for the past year after the mindfulness training program?
12	Any questions or comments you would like to make?	

**Table 3:** **The interview questions for the Familiar Support Persons (FSPs) at post-intervention (T2) and 1-year follow-up (T3)**.

**Qn**	**Post-intervention (T2)–30 min**	**1-year follow-up (T3)–20 min**
1	What has it been like supporting (first name of the MCI participant)?	
	I really appreciate you sharing your views.	
2	From your personal perspective, how has the mindfulness training program experience been for you?	
	How about your perspective on the program experience as a familiar support person?	
3	Can you please describe how has attending the program influenced you personally?	How has mindfulness influenced you in the past year?
4	How can we improve the mindfulness training program experience for future familiar support persons?	
5	Can you please describe how (first name of the MCI participant) has practiced mindfulness?	Can you please describe how (first name of the MCI participant) has practiced mindfulness in the past year?
	How do you think (first name of the MCI participant) has used mindfulness in his/her day-to-day life?	How do you think (first name of the MCI participant) has used mindfulness in his/her day-to-day life?
6	From your perspective, how has mindfulness influenced (first name of the MCI participant)'s health and life?	From your perspective, how has mindfulness influenced (first name of the MCI participant)'s health and life in the past year?
	How useful do you think mindfulness has been for him/her?	How useful do you think mindfulness has been for him/her overall?
	*[Interviewer's prompts: cognitive function, psychological health, functional abilities & quality of life]*	*[Interviewer's prompts: cognitive function, psychological health, functional abilities and quality of life]*
7	What do you think about your role in supporting (first name of the MCI participant) in mindfulness practice during and outside the program?	What do you think about your role in supporting (first name of the MCI participant) in mindfulness practice in the past year?
8	Any questions or comments you would like to make?	

## Stepwise procedures

Altogether, there are a total of 3 data collection sessions in this study, which will be held at any of the Monash University campuses or a public venue such as library and community club that is convenient to the MCI participant and his/her FSP. The MCI participant will be administered the MoCA, the 21-item DASS-21, the 14-item FMI and the 12-item MAQ by researcher (WW) during these data collection sessions at three time points:

- Pre-intervention (T1);
- Post-intervention (T2); and
- One-year follow-up (T3).

These measurements should take about 30 min each session and will be conducted to compare the effects of mindfulness on the outcomes measures across these time frames and to determine whether any changes are sustained over time. By incorporating a 1-year follow-up into the study design, it could more effectively measure the longitudinal effects of mindfulness practice on persons with MCI over time. A semi-structured interview with the MCI participant that should take about 30 min, will be conducted by researcher (WW) at T2 and T3 after he/she has completed the above-mentioned cognitive assessment and questionnaires/scales.

The nature of the participants' MCI condition could possibly fluctuate between the time frames of provision of written informed consent (with their capacity to consent as confirmed by their FSP) and the 1-year follow-up period. In other words, their MCI condition could remain stable, improve or deteriorate to AD during the study duration. In the event that the MCI participant's MoCA score suggests severe cognitive impairment, his/her continued study participation will be reviewed and the referral to his/her GP or treating health professional for follow-up will be discussed with him/her and his/her FSP. Likewise, if the MCI participant reports a clinically significant DASS-21 score, the research team will contact him/her to ensure his/her well-being and seek his/her permission to inform his/her FSP and forward the score to his/her GP or treating health professional for follow-up. Any clinically significant information about the MCI participants collected from their study participation, will be stored in their health records with the participating CDAMS sites or communicated to their GP or treating health professional.

While the administration of the cognitive assessment and questionnaires/scales is in progress with the MCI participant during each of the three data collection sessions, the FSP will provide information about the MCI participant by completing the B-ADL Scale and the DHL questionnaire on his/her own. During T2 and T3, the FSP will be asked to attend a semi-structured interview that will take up to 30 min after the interview session with the MCI participant is completed. In sum, the FSP's study involvement duration during T1 is about 30 min, and during T2 and T3 is about an hour each. Should the FSP be unavailable to attend T2 and/or T3, he/she can return the completed B-ADL Scales and DHL questionnaires using reply paid return envelopes provided, and arrangements can be made for the interviews with the FSP to be conducted over the phone.

**Figure 1:**
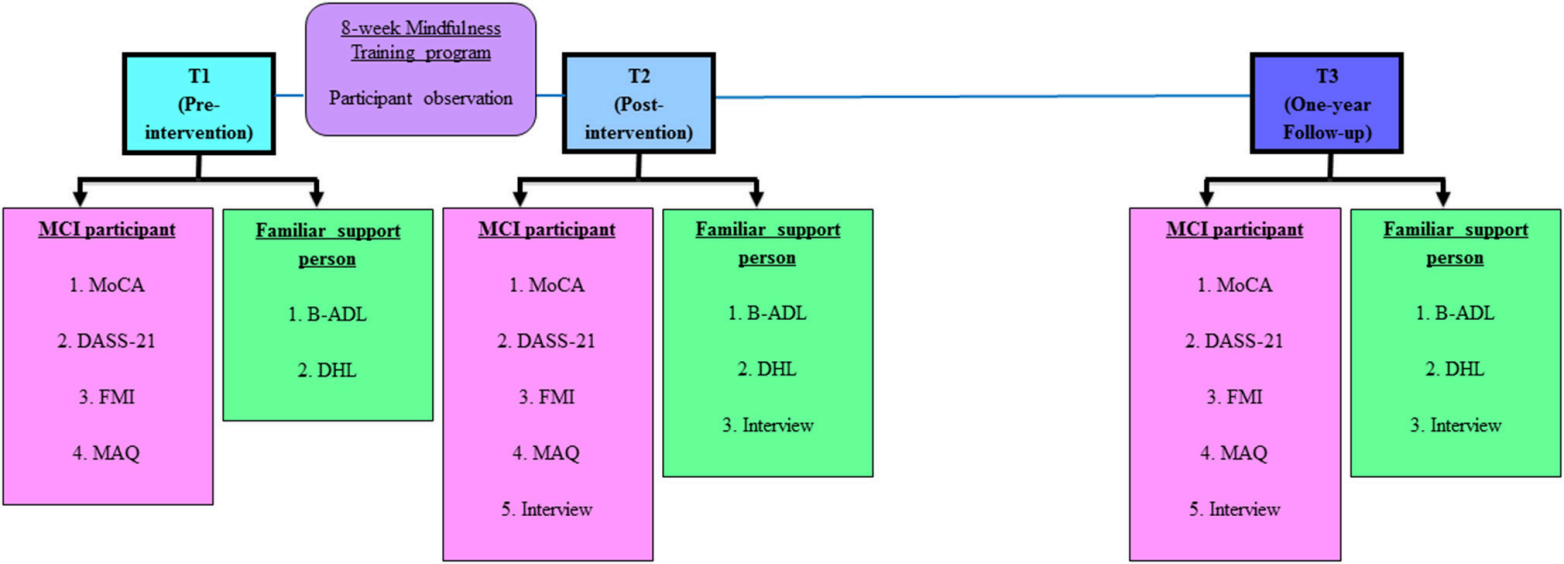
**Stages of study protocol**. Abbreviations (in alphabetical order): B-ADL, Bayer Activities of Daily Living (25-item); DASS 21, Depression Anxiety Stress Scales (21-item); DHL, Demographic, Health and Lifestyle questionnaire; FMI, Freiburg Mindfulness Inventory (14-item); MAQ, Mindfulness Adherence Questionnaire (12-item); MCI, Mild Cognitive Impairment; MoCA, Montreal Cognitive Assessment.

For an overview of this study, the stages of the study protocol are illustrated in Figure 1.

## Anticipated results

Repeated measures analysis of variance will be used to test the relative effects of formal and informal mindfulness practices on changes in the following dependent variables, namely cognitive function, psychological health, mindfulness, and ADL functioning from pre- to post-intervention and from post-intervention to 1-year follow-up, with an experiment-wise error rate of 0.05. Normally, each ANOVA should be evaluated with an adjusted (for e.g., Bonferroni correction) decision-wise error rate for possible inflation of Type I error. However, Type I error rate will not be adjusted for these analyses, on the grounds that this is an exploratory study of which any possible effects should be examined by further research. Regression analysis will be used to determine the predictive values of measures of formal and informal mindfulness practices on the changes in the same dependent variables (cognitive function, psychological health, mindfulness, and ADL functioning). Age, gender, and education will be entered into the regression analysis to identify possible moderating effects. The MCI participants' demographics, health and lifestyle information will be examined in relation to the outcome measures in order to interpret the results in context. However, possible trends instead of statistically significant results could be determined from the quantitative analysis. As such, the mixed-methods design of this study enables the qualitative findings to complement the quantitative results in greater depth so that their findings could inform each other and be integrated to better address the research questions. The IBM Statistical Package for the Social Sciences software package will be used for the statistical analysis of the quantitative data.

**Figure 2:**
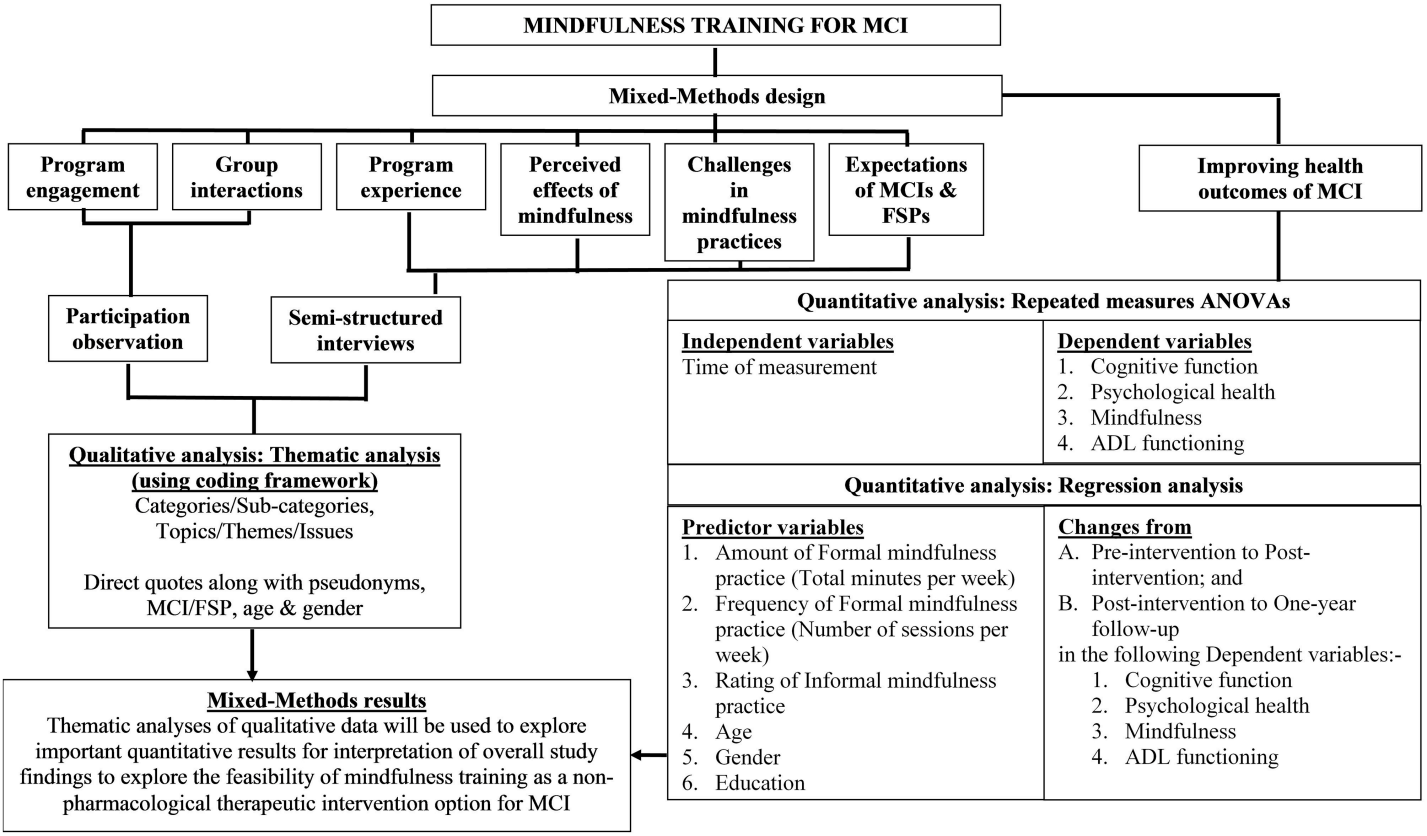
**Framework of the presentation of qualitative and quantitative data**. ADl, Activities of Daily Living; ANOVA, Analysis of variance; FSP, Familiar Support Person; MCI, Mild Cognitive Impairment.

Qualitative data analyses by researchers (WW) and Jan Coles (JC) will entail the investigation of the participant observational field notes and the semi-structured interview transcripts. To safeguard privacy, any identifiable information such as the name of the MCI participant, FSP, CDAMS, health service, GP and treating health professional in the audio recording of the interviews will be coded (but re-identifiable by the researchers for qualitative data analyses) in the interview transcripts. The QSR International NVivo software will be used to perform thematic analyses for the identification of important patterns/themes. The iterative (repeating) and inductive (bottom up) process of thematic analysis in the qualitative data analysis phase breaks the data into “smaller chunks” and assigns names or “codes” that summarize the idea within the data chunk (Hansen, 2006; Liamputtong, 2013). The codes are then refined by comparing, sorting and reflecting on the meaning and subsequently developed into “themes”^1^. A coding framework will be developed to provide definitions of the codes and maximize coherence among the codes for coding the qualitative data and generating themes or categories (Creswell, 2014). For inter-coder reliability, codes developed by researchers (WW and JC) will be cross-checked for agreement in coding with the same or similar code in order to maintain consistency in coding (Creswell, 2014). The framework of the presentation of both qualitative and quantitative data is illustrated in Figure 2.

The MCI participants and FSPs have the option to receive an email containing a summary of the non-identifiable group results when this study is completed. The findings from this study will be disseminated through peer-reviewed academic journal publications and presented at national and international seminars and conferences. In any publication and presentation, the information will be presented in non-identifiable form to protect the privacy of the MCI participants and their FSP.

It is expected that the mindfulness training intervention will proactively engage the MCI participants early and yield the practical application of improving their cognitive function such as attention and memory if they maintain mindfulness practice over time. There may be a potential benefit of delaying the progression of MCI to AD but this will not be assessed in this study. In addition, other expected improvements in psychological health, mindfulness, and in functional abilities of ADL may also indicate better health outcomes of mindfulness for MCI. This study will contribute toward the clinical research of MCI and advance evidence-based knowledge about the effects of long-term mindfulness practice as a promising and feasible non-pharmacological therapeutic approach for persons with MCI. This could potentially translate to downstream significance of better quality of life and higher work productivity of persons with MCI and their FSP, and a reduced chronic disease burden on families of persons with MCI and the healthcare system. If effective, “partnerships could be eventually forged with the Alzheimer's Australia and memory clinics/services such as the CDAMS to deliver the customized group mindfulness training program to the target population of persons with MCI and their FSP” (Wong et al., 2015).

The study findings could open a potential future research direction to explore the use of biomarkers such as beta-amyloid and tau proteins to monitor the longitudinal effects of mindfulness practice on the cognitive and functional progression of persons with MCI and the conversion rate from MCI to AD.

## Potential pitfalls and counteracting measures

This study was originally designed as a randomized controlled trial using pre- and post-intervention design that will assign persons who have been clinically diagnosed with MCI to either the 8-week mindfulness training intervention group or the “standard care” control group. In the original randomized controlled trial design, the estimated total sample size is 166–83 MCI participants in the intervention group and 83 in the control group, in order to achieve 80% power for a two-tailed test at the alpha level of 0.05 with a medium effect size of 0.50. The minimum sample size required to detect a clinically significant difference is 68 participants–34 in each of the two groups. This design was approved by the Melbourne Health Human Research Ethics Committee. However, due to the challenges in the study recruitment of MCI participants, changes had to be made to the randomized controlled trial study design to that of a pilot study that would explore the feasibility of the mindfulness training intervention for future research. Consequently, the revised target sample size is about 20 MCI participants. An estimation of 80 interviews conducted with 20 MCI participants and 20 FSPs at T1 and T2, and coupled with participant observation of the mindfulness program produce rich qualitative data and provide important participant perspectives such as learning and maintaining a new behavior, that could yield significant contributions to the field (Morse, 2000; Creswell, 2007), especially in this sub-clinical population that is under explored. The findings would be relevant to better understand this population, help future study recruitment, and shed insights to further research studies with this population. This revised study design was also approved by the Melbourne Health Human Research Ethics Committee.

As the age profile of the MCI participants is senior (at least 60 years old), program attendance and participation might be affected by various circumstances such as clinically significant scores of cognitive impairment measured by MoCA and of psychological health measured by DASS-21, conversion to dementia such as Alzheimer's disease, and pre-existing medical conditions. The research team will review the participants' continued study participation and notify their GP or treating health professional for follow-up after discussion with them.

The MCI participants are required to attend at least six of the eight sessions to have satisfactorily completed the mindfulness training program. The MCI participants and FSPs are provided with the program manual and the mindfulness practice audio CD, and researcher (WW) contacts the MCI participants weekly during the program duration to discuss any questions, concerns and issues that they might have. WW will also inform the outline of the weekly module to those who have missed the session.

To encourage the MCI participants to do their mindfulness practices and obtain their monthly practice record forms, researcher (WW) will contact them on a monthly basis after their completion of the mindfulness training program. However, due to the extent of cognitive impairment of certain MCI participants, particularly for those who are not living with their FSP, WW might have to contact them more frequently to better support them. Such study rigor might impose some inconvenience on these MCI participants. Thus, WW will check with them on their comfortable frequency of post-intervention communications such as fortnightly phone calls.

The MCI participants and FSPs are free to withdraw consent from the study at any stage by informing the research team which will still include the previous data and information that are already collected from them in the data analyses to form part of the study findings.

While the study findings from this reasonably homogeneous sample may be potentially positive, however, due to the small sample size, it is not likely to reach statistical significance. As such, the study will also share the similar study limitation of small sample sizes in previous studies and its results may not be generalizable to the broader community.

## Author contributions

WW developed and drafted this study protocol in consultation with JC, CH and subject matter experts; provided inputs to the design of the customized group MCI mindfulness training program intervention and the development of the program manual and the MAQ; adapted the DHL questionnaire; and prepared the human research ethics and research governance applications. CH who is an acclaimed mindfulness expert, was involved in the overall research design and selection of outcome measures, the design and facilitation of the group mindfulness training program intervention; and the development of the MAQ and program manual. RC who is also an acclaimed mindfulness expert, was involved in the design and facilitation of the group mindfulness training program intervention; and the development of the MAQ and program manual. JC who is the Chief Researcher of this study, was involved in the overall research design and selection of appropriate qualitative research questions, mixed-methods research methodologies, outcome measures, and semi-structured interview questions.

## Funding

This study was funded using the General Operating Fund and Consultancy Fund of the Department of General Practice, Faculty of Medicine, Nursing and Health Sciences, Monash University.

### Conflict of interest statement

As authors CH, RC are mindfulness experts who designed the customized group Mild Cognitive Impairment mindfulness training program and developed the Mindfulness Adherence Questionnaire, there might be a perceived conflict of interest. However, the data analysis and interpretation will be performed in a fair and appropriate way and totally independent from both authors involved in the intervention.

## Footnotes

^1^(2006). 10 questions to help you make sense of qualitative research. England: Public Health Resource Unit.

## Abbreviations

AD: Alzheimer's Disease
ADL: Activities of Daily Living
B-ADL: Bayer Activities of Daily Living
CDAMS: Cognitive Dementia and Memory Service
CH: Craig Hassed (author)
DASS-21: 21-item Depression Anxiety Stress Scales
DHL: Demographic Health and Lifestyle
e.g.: For example
FMI: Freiburg Mindfulness Inventory
FSP: Familiar Support Person
GP: General Practitioner
JC: Jan Coles (author)
MAQ: Mindfulness Adherence Questionnaire
MBSR: Mindfulness-Based Stress Reduction
MCI: Mild Cognitive Impairment
MoCA: Montreal Cognitive Assessment
RC: Richard Chambers (author)
WW: Wee Ping Wong (author).
