# Supplementary material for: The Effects of Mindfulness on Persons with Mild Cognitive Impairment: Protocol for a Mixed-Methods Longitudinal Study
Source: Front Aging Neurosci. 2016 Jun 28;8:156. doi: 10.3389/fnagi.2016.00156 (PMC4923201; doi:10.3389/fnagi.2016.00156)
Supplement: Supplementary file 1 [file DataSheet1.PDF]

Supplementary material

The Effects of Mindfulness on Persons With Mild Cognitive Impairment: Protocol for a Mixed-Methods Longitudinal Study

Wee Ping Wong*, Craig Hassed, Richard Chambers, Jan Coles

*** Correspondence:** Ping Wong: ping.wong@monash.edu

Mindfulness Adherence Questionnaire (MAQ)

How often was each of the following statements true for you during the **PAST WEEK**?
Please circle the appropriate number on each line.

1. Over the past week, I practiced mindfulness meditation (formal practice) for __________ (number of times) and __________ (average minutes for each time).
If you have not practiced mindfulness meditation over the past week, then please skip to the questions on the next page.

FORMAL MINDFULNESS PRACTICE	Never	Some-times	Often	Most of the time
**2. When meditating, how much of the time was your attention on what it should have been?**	1	2	3	4
**3. When meditating, how much of the time were you distracted?**	1	2	3	4
**4. When meditating, were you becoming more aware of when your mind is distracted?**	1	2	3	4
**5. When meditating, how much of the time were you adopting an accepting attitude to what you were experiencing?**	1	2	3	4

Please turn page over.

How often was each of the following statements true for you during the **PAST WEEK**?
Please circle the appropriate number on each line.

**INFORMAL MINDFULNESS PRACTICE**	**Never**	**Some-times**	**Often**	**Most of the time**
**6. I practiced being mindful in my day-to-day activities such as eating, walking, listening, talking, doing chores etc.**	1	2	3	4
**7. I practiced being mindful during emotional situations such as happiness, joy, love, anger, stress and anxiety.**	1	2	3	4
**8. During day-to-day life, I brought my attention / awareness back to what I was doing in the present moment.**	1	2	3	4
**9. I consciously practiced acceptance, letting go, curiosity, gentleness and being in the present moment.**	1	2	3	4
**10. I practiced being mindful while Iearning new things, or gathering new knowledge or information.**	1	2	3	4
**11. I practiced being mindful during problem-solving situations.**	1	2	3	4
**12. I practiced being mindful while participating in my hobbies and interests.**	1	2	3	4
