# Supplementary material for: The Effects of Mindfulness on Persons with Mild Cognitive Impairment: Protocol for a Mixed-Methods Longitudinal Study
Source: Front Aging Neurosci. 2016 Jun 28;8:156. doi: 10.3389/fnagi.2016.00156 (PMC4923201; doi:10.3389/fnagi.2016.00156)
Supplement: Supplementary file 2 [file DataSheet2.PDF]

Supplementary material

The Effects of Mindfulness on Persons With Mild Cognitive Impairment: Protocol for a Mixed-Methods Longitudinal Study

Wee Ping Wong*, Craig Hassed, Richard Chambers, Jan Coles

*** Correspondence:** Ping Wong: ping.wong@monash.edu

Demographic, Health & Lifestyle (DHL) Questionnaire

Q1: Participant's present marital status:

A. Married (registered)
B. De facto relationship
C. Separated
D. Divorced
E. Widowed
F. Never married

Q2: Your relationship to the participant:

A. Husband
B. Wife
C. De facto partner
D. Separated
E. Ex-husband
F. Ex-wife
G. Others (please specify): __________

Q3: When did you start providing support for the participant? __________ MM/YYYY

Q4: How often in total do you provide support to the participant?

A. Every day
B. Several times a week
C. Once a week
D. Once every few weeks
E. Less often

Q5: How much time do you usually spend providing such support to the participant on each occasion?

A. All day and night
B. All day
C. All night
D. Several hours
E. About an hour

Q6: Do you live with the participant? Yes/ No

Health

Q7: In the PAST THREE YEARS, has the participant been diagnosed or treated for: *(Circle accordingly)*
Diabetes (high blood sugar), Heart disease (including heart attack, angina), Hypertension (high blood pressure), High cholesterol, Stroke, Parkinson's disease, Alzheimer's disease or dementia, Cancer, Depression, Anxiety/ Nervous disorder,
Others ____

Q8: When was the participant diagnosed with Mild Cognitive Impairment (MCI) by which memory service/ clinic (e.g. CDAMS)? What is the prescribed treatment?
Date of diagnosis : ______________________________ (DD/MM/YYYY)
Place of diagnosis : ____
Prescribed treatment :____
____
____
____
____
____

Q9: Has the participant had any medical operations or procedures in the PAST THREE YEARS? Yes (please specify)/ No
____
____
____
____
____

Q10: Please list the type of medications prescribed by a doctor, medications/ vitamins/ minerals/ supplements/ herbal therapies bought without a prescription at the chemist, supermarket or health food shop and other alternative therapies (e.g. Chinese medicines) that the participant has been taking during the PAST FOUR WEEKS?
____
____
____
____
____

Physical & Social Activity

Q11: How many hours EACH DAY does the participant typically spend sitting down while at home, at work, getting from place to place or during spare time, doing things like visiting friends, driving, reading, watching television or working at a desk or computer?
_______ hours

Q12: How many times did the participant do each type of activity LAST WEEK?
Only count the number of times when the activity lasted for 10 minutes or more.
(If he/she did **not** do an activity, please write '0' in the box.)

A. **Walking briskly** (for recreation or exercise, or to get from place to place) _________ times
B. **Moderate leisure activity** (like social tennis, moderate exercise classes, recreational swimming, dancing) _________ times
C. **Vigorous leisure activity** (that makes him/her breathe harder or puff and pant like aerobics, competitive sport, vigorous cycling, running, swimming) _________ times
D. **Vigorous household or garden chores** (that make him/her breathe harder or puff and pant) _________ times
E. **Socializing with people** (social interactions with family, relatives, friends, support/ network/ spiritual groups) _________ times

Q13: If you add up all the times the participant spent in each activity LAST WEEK, how much time did he/she spend ALTOGETHER doing each type of activity?
(If he/she did **not** do an activity, please write '0' in the box.)

A. **Walking briskly** (for recreation or exercise, or to get from place to place) ______ hours ______ minutes
B. **Moderate leisure activity** (like social tennis, moderate exercise classes, recreational swimming, dancing) ______ hours ______ minutes
C. **Vigorous leisure activity** (that makes him/her breathe harder or puff and pant like aerobics, competitive sport, vigorous cycling, running, swimming) ______ hours ______ minutes
D. **Vigorous household or garden chores** (that make him/her breathe harder or puff and pant) ______ hours ______ minutes
E. **Socializing with people** (social interactions with family, relatives, friends, support/ network/ spiritual groups) ______ hours ______ minutes

Lifestyle Activities

Q14: How often over the PAST ONE YEAR the participant had participated in each of the following activities? Please indicate the corresponding letters on the line accordingly.

A = Never or less than once a month
B = Once a month
C = 2 to 3 times a month
D = Once a week
E = 2 to 3 times a week
F = Every day

1. Crossword puzzles ______
2. Taking courses/ classes ______
3. Reading books ______
4. Drawing ______
5. Singing or playing music ______
6. Reading newspapers ______
7. Talking about local/ national issues ______
8. Discussing politics ______
9. Playing cards/ games ______
10. Assisting family or friends ______

11. Volunteer work _____
12. Participating in church/ club/ organizations _____
13. Sewing/ Mending/ Fixing/ Decorating/ Building _____
14. Cooking _____
15. Going to plays or concerts _____
16. Attending religious services _____
17. Gardening _____
18. Going to movies _____
19. Listening to radio _____
20. Listening to music _____
21. Watching television _____
22. Others (please specify, for example using a computer for word processing or email & Internet) _____

Dietary Intake

Q15: During the PAST ONE YEAR, how many times did the participant eat wholemeal or wholegrain foods per week?

A. 6 or more times per week
B. 4 to 5 times per week
C. 2 to 3 times per week
D. Once a week
E. Less than once a week

Q16: During the PAST ONE YEAR, how many times did the participant eat cereals (e.g. pasta, rice, noodles, cous cous) as part of his/her main meal per week?

A. 5 or more times per week
B. 3 to 4 times per week
C. 1 to 2 times per week
D. Rarely
E. Never

Q17: During the PAST ONE YEAR, how many serves of vegetables did the participant eat in a typical day? *(One serve is equal to half a cup of cooked vegetables or a cup of salad)*

A. 5 or more serves
B. 3 to 4 serves
C. 2 serves
D. 1 serve
E. Less than 1 serve or none

Q18: During the PAST ONE YEAR, how many pieces of fruit did the participant eat in a typical day? *(One piece is ½ cup of diced fruit, berries or grapes)*

A. 2 or more pieces
B. 1 piece
C. Less than 1 piece

Q19: During the PAST ONE YEAR, how many times did the participant eat legumes (e.g. chickpeas, baked beans, 3 bean mix, lentils, split peas, dried beans)?

A. 2 or more times per week

B. Once a week
C. Once a fortnight
D. Rarely
E. Never

Q20: During the PAST ONE YEAR, how many times did the participant eat raw nuts or seeds (e.g. pepitas, sunflower seeds, linseed) per week?

A. 5 or more times per week
B. 3 to 4 times per week
C. 1 to 2 times per week
D. Less than once a week
E. Never

Q21: During the PAST ONE YEAR, how many times did the participant eat fish or take fish oil supplements per week?

A. 2 or more times per week
B. Once a week
C. Less than once a week
D. Never

Q22: During the PAST ONE YEAR, how many times did the participant eat poultry/ meat per week?

A. 5 or more times per week
B. 3 to 4 times per week
C. 1 to 2 times per week
D. Less than once a week
E. Never

Q23: When cooking meat/ fish during the PAST ONE YEAR, which of the following cooking fats was normally used to prepare meals for the participant?

A. Butter
B. Solid frying fat
C. Vegetable oil (e.g. coconut, palm)
D. Monounsaturated oils (e.g. olive, canola, pecan, almond, peanut) or Polyunsaturated oils (e.g. corn, soy, cotton seed, safflower, sunflower, flaxseed, walnut, fish)
E. Spray oil
F. Cooking fat was not used

Q24: When having milk, yogurt or cheese during the PAST ONE YEAR, how often did the participant consume or use reduced fat or skimmed products?

A. Always
B. Usually
C. Occasionally
D. Rarely
E. Never

Q25: During the PAST ONE YEAR, how many times did the participant eat pastries, cakes, sweet biscuits or croissants per week?

A. 6 or more times per week
B. 4 to 5 times per week

C. 3 times per week
D. 1 to 2 times per week
E. Never or Less than once a week

Alcohol Consumption

Q26: How often does the participant usually drink alcohol? *(Mark one only)*

A. He/she has never drunk alcohol in his/her life
B. He/she never drinks alcohol, but he/she has in the past
C. He/she drinks rarely
D. Less than once a week
E. On 1 or 2 days a week
F. On 3 or 4 days a week
G. On 5 or 6 days a week
H. Every day

Q27: On a day when the participant drinks alcohol, how many standard drinks (1 standard drink = 1 pot of beer, 1 nip of spirits, 1 medium glass of wine) does he/she usually have? *(Mark one only)*

A. 1 or 2 drinks per day
B. 3 or 4 drinks per day
C. 5 to 8 drinks per day
D. 9 or more drinks per day

Smoking

Q28: How often does the participant currently smoke cigarettes or any tobacco products? *(Mark one only)*

A. Daily
B. At least weekly (but not daily)
C. Less often than weekly
D. Not at all

Q29: If the participant smokes daily, on average how many cigarettes does he/she smoke EACH DAY? _____ cigarettes per day

Q30: If the participant used to smoke daily in the past, at what age did he/she finally stop smoking DAILY? _____ years old

Demographic Information

Q31: We would like to know YOUR and THE PARTICIPANT'S current main occupation:
(Mark one in each column)

	Self	Participant
**Manager or Administrator**		

(e.g. magistrate, farm manager, media producer, school principal)		
**Professional**<br>(e.g. registered nurse, allied health professional, teacher, artist)		
**Associate Professional**<br>(e.g. office manager, branch manager, shop manager, retail buyer, youth worker, police officer)		
**Tradesperson or related worker**<br>(e.g. cook, dressmaker, hairdresser, gardener, florist)		
**Advanced clerical or service worker**<br>(e.g. credit officer, radio despatcher, personal assistant, flight attendant, law clerk)		
**Intermediate clerical, sales or service worker**<br>(e.g. accounts clerk, checkout supervisor, data entry operator, child care worker, nursing assistant, hospitality worker)		
**Intermediate production or transport worker**<br>(e.g. machine operator, bus driver)		
**Elementary clerical, sales or service worker**<br>(e.g. filing/ mail clerk, parking inspector, sales assistant, telemarketer, housekeeper)		
**Laborer or related worker**<br>(e.g. cleaner, factory worker, kitchen hand, fast food cook)		
**No paid job**		
**Retired**<br>- Please indicate year of retirement on the respective line.	________	________
**Gave up paid work**<br>- Please indicate year of complete giving up of work on the respective line.	________	________

Q32: What is the participant's highest educational qualification? *(Mark one only)*

**Primary school**	
**Secondary school**	
**Senior Secondary school**	
**Vocational Education and Training (VET)**	
**Bachelor degree**	
**Postgraduate Diploma**	
**Master degree**	
**PhD degree**	
**Others (please specify)**	

Q33: What is the participant's RESIDENTIAL SUBURB in Victoria, Australia? (where he/she lives)

____

Q34: What is the participant's POSTAL ADDRESS? (where correspondences could be mailed)

____

____

____

Q35: What is your POSTAL ADDRESS (if different from the participant)?

____
____
____

Q36: What is the participant's EMAIL ADDRESS (if he/she has one)?

____

Q37: What is your EMAIL ADDRESS?

____

Please let us know your new postal address if you move or change your email address or telephone numbers.

Q38: What is the participant's date of birth? __________ DD/MM/YYYY

Q39: What is your year of birth? __________ YYYY

Q40: In case of emergency, please provide the name and contact details of an emergency contact person:

Name: ____
Relationship to Participant: ____
Mobile phone: ____
Home phone: ____

Q41: Please provide the name and contact details of the participant's General Practitioner/ treating health professional:

Name: ____
Health Service: ____
Address: ____
Phone: ____

Have we missed anything?

If there is ANYTHING else you would like to tell us about the participant, please write on the lines below.

____
____
____
____
____
____
____
____

~*~*~*~*~* THANK YOU VERY MUCH! MUCH APPRECIATED! ~*~*~*~*~*
